# IoT and Interpretable Machine Learning Based Framework for Disease Prediction in Pearl Millet

**DOI:** 10.3390/s21165386

**Published:** 2021-08-09

**Authors:** Nidhi Kundu, Geeta Rani, Vijaypal Singh Dhaka, Kalpit Gupta, Siddaiah Chandra Nayak, Sahil Verma, Muhammad Fazal Ijaz, Marcin Woźniak

**Affiliations:** 1 Department of Computer and Communication Engineering, Manipal University Jaipur, Jaipur 303007, India; kundu.nidhi1990@gmail.com (N.K.); vijaypalsingh.dhaka@jaipur.manipal.edu (V.S.D.); kalpit.189201045@muj.manipal.edu (K.G.); 2 ICAR DOS in Biotechnology, University of Mysore Manasagangotri, Mysore 570005, India; moonnayak@gmail.com; 3 Department of Computer Science and Engineering, Chandigarh University, Mohali 140413, India; sahilverma@ieee.org; 4 Department of Intelligent Mechatronics Engineering, Sejong University, Seoul 05006, Korea; 5 Faculty of Applied Mathematics, Silesian University of Technology, 44-100 Gliwice, Poland; marcin.wozniak@polsl.pl

**Keywords:** machine learning, interpretable, context-aware, deep learning, IoT

## Abstract

Decrease in crop yield and degradation in product quality due to plant diseases such as rust and blast in pearl millet is the cause of concern for farmers and the agriculture industry. The stipulation of expert advice for disease identification is also a challenge for the farmers. The traditional techniques adopted for plant disease detection require more human intervention, are unhandy for farmers, and have a high cost of deployment, operation, and maintenance. Therefore, there is a requirement for automating plant disease detection and classification. Deep learning and IoT-based solutions are proposed in the literature for plant disease detection and classification. However, there is a huge scope to develop low-cost systems by integrating these techniques for data collection, feature visualization, and disease detection. This research aims to develop the ‘Automatic and Intelligent Data Collector and Classifier’ framework by integrating IoT and deep learning. The framework automatically collects the imagery and parametric data from the pearl millet farmland at ICAR, Mysore, India. It automatically sends the collected data to the cloud server and the Raspberry Pi. The ‘Custom-Net’ model designed as a part of this research is deployed on the cloud server. It collaborates with the Raspberry Pi to precisely predict the blast and rust diseases in pearl millet. Moreover, the Grad-CAM is employed to visualize the features extracted by the ‘Custom-Net’. Furthermore, the impact of transfer learning on the ‘Custom-Net’ and state-of-the-art models viz. Inception ResNet-V2, Inception-V3, ResNet-50, VGG-16, and VGG-19 is shown in this manuscript. Based on the experimental results, and features visualization by Grad-CAM, it is observed that the ‘Custom-Net’ extracts the relevant features and the transfer learning improves the extraction of relevant features. Additionally, the ‘Custom-Net’ model reports a classification accuracy of 98.78% that is equivalent to state-of-the-art models viz. Inception ResNet-V2, Inception-V3, ResNet-50, VGG-16, and VGG-19. Although the classification of ‘Custom-Net’ is comparable to state-of-the-art models, it is effective in reducing the training time by 86.67%. It makes the model more suitable for automating disease detection. This proves that the proposed model is effective in providing a low-cost and handy tool for farmers to improve crop yield and product quality.

## 1. Introduction

The traditional systems of farming focus on meeting the dietary requirements of people and domestic animals. Therefore, the farmers used to grow more nutritious cereals such as millets and sorghum rather than high-yielding grains such as rice and wheat. With the commercialization of agriculture, the farmers have shifted their interest towards high crop yields that can fulfill their dietary and financial requirements. This shift has increased the burden of malnutrition, causing undernourishment and micronutrient deficiencies [1]. Therefore, there is a need to implement the precision system of agriculture that improves the yield and the quality of highly nutritious crops.

The prime minister recognized millets as a treasure of nutrition and commended for a call to start a millet revolution in India. He has declared millets as the ‘Nutri Cereals’ for production, consumption, and trade points [2]. In addition, the Union Ministry for Human Resource Development (MHRD) [3] has requested states to include Millet in Mid-day meals served in schools. Moreover, a continuous decrease in the yield of common crops such as wheat, rice, groundnut, and maize [4,5] has attracted farmers to grow pearl millet. Pearl millet is resilient to climate issues due to its less water demand of 200 to 600 mm, stability at high temperatures, and drought-prone ability. Therefore, millets with their ‘Nutri Cereals’ capability can be tapped for food security in the future. To meet this rising demand, the farmers started the use of fertilizers, pesticides, and controlled irrigation. This has increased the global crop yield of pearl millet over the past 50 years [6,7]. As per the Project Coordinator, a review report published by the Directorate of Millets Development, 2020, the pearl millet covers 6.93 million hectares of land. The average production of 8.61 million tons was reported during 2018–2020 [5].

The productivity and quality of pearl millet are adversely affected by plant diseases such as blast and rust [5]. These diseases put a substantial threat to food security and harm the economy of farmers [8,9]. Therefore, it is mandatory to introduce a system for the detection of diseases in crop plants.

Context-aware and interpretable machine learning (ML) and deep learning (DL) have gained remarkable attention in human health monitoring [10,11,12,13,14,15,16], crop health monitoring, and yield prediction [17]. These models are effective to give automatic, accurate, and quick systems for plant disease detection and classification. Several models of ML and DL are found effective and precise in disease detection and classification [18]. However, as per the discussion given in [19,20], the convolutional neural network (CNN) outperforms the machine learning (ML) models due to their potential in automatic feature extraction. However, CNN models demand a huge and labeled dataset for training. The collection of the dataset and correct labeling of a disease in a vast dataset is challenging as it requires time and effort from the experts.

The frameworks based on the Internet of Things (IoT) have proven their acceptance in automating data collection, data storage, and processing of collected datasets to make real-time predictions [10,11,21]. The adequate use of drone, camera, and sensors significantly reduce the time and cost of labor.

Furthermore, the advancements in deep transfer learning bring down the requirement for the vast dataset [22]. In this approach, the model is initially trained on an extensive dataset that is not necessarily labelled. At this stage, the model learns about the low-level features such as texture, pixel intensity, marking of boundaries, etc. The weights of this trained model are saved and utilized for further training the model with the dataset comprising labelled samples [22].

The potential of IoT in data collection, data storage, quick processing, and efficacy of interpretable ML, DL, and transfer learning techniques in object detection, classification, visualization, and pattern matching even with the small-sized labelled dataset [19,23,24] motivated the authors to employ the integration of these techniques for developing a framework for detection of disease in pearl millet.

In this manuscript, the authors propose an IoT and interpretable deep transfer learning-based framework, ‘Automatic and Intelligent Data Collector and Classifier’ (AIDCC), for the detection and classification of diseases viz. rust and blast [25] in pearl millet.

The significant contributions of this manuscript are as follows:

1. Highlighting the need to automate the detection of diseases in the underexplored crop ‘pearl millet’.
2. Automatic collection of the real-time datasets by the IoT system fixed at the farmlands of pearl millet.
3. Developing the IoT and deep transfer learning-based framework for detection and classification of diseases in pearl millet.
4. Presenting the comparative analysis of the proposed framework and the systems available in the literature to detect and classify plant diseases.

## 2. Related Works

The extensive study of literature in plant disease detection and classification gives insights into the techniques employed to collect datasets, pre-processing, disease detection, classification, and visualization.

In the traditional approaches, the farmers manually detect diseased and healthy plants [26]. These approaches lack in the tracking of the essential parameters such as soil type, humidity, temperature, amount of macro and micronutrients in the soil, and nutrient requirements of the crop plant at different stages of its growth and maturity. Moreover, the traditional approaches are time-consuming and need a lot of human effort. Furthermore, the farmers need advice from experts for the correct diagnosis of diseases in crop plants. 

The applications of IoT, computer vision, ML, DL, and deep transfer learning have streamlined the automation of plant disease detection and classification [27,28]. In this line of research, the authors proposed IoT and ML models for data capturing and disease prediction [29]. They used the drone for capturing the images over a large area in less time. They applied the support vector machine (SVM) for the classification of diseases in rice crops. However, their system did not consider the on-demand capturing of images for real-time monitoring and prediction.

Furthermore, the authors in [30] utilized the potential of IoT to categorize the healthy and diseased leaves. They anchored the sensors for monitoring of soil quality, temperature, and humidity. They used the camera to capture the images of crop plants. The authors established the interface of sensors and a camera with the Raspberry Pi to store and process the captured data for real-time predictions. They employed the K-means algorithm for clustering images followed by masking of pixels to detect whether the leaf is diseased or healthy.

The authors in [31,32] stated that DL techniques are effective in the early detection of crop diseases. They recommended the use of these techniques to overcome the limitations of traditional approaches. The research works presented in [24,27,32,33,34,35,36,37,38] and [39,40,41,42,43,44,45,46] introduced deep learning (DL) models for the detection and classification of plant diseases. Furthermore, Mohammed Brahimi claimed the supremacy of deep transfer learning over deep learning [27].

The authors in [39] collected a dataset of 36,258 images from the AI challenger [47]. However, the dataset comprised images of poor visual quality. They employed the ResNet model on the collected dataset and reported an accuracy of 93.96%.

The works presented by the authors in [19,48,49] highlighted the importance of collecting imagery datasets and employing an appropriate DL model on the collected dataset for the detection and classification of plant diseases. They also focused on integrating DL models with the IoT systems comprising sensors, a drone, a camera, etc. They claimed that these integrated systems effectively minimize human efforts and reduce the time required for different agricultural practices. These systems are capable of gathering real-time information from farms and quick processing of the collected datasets to predict plant diseases.

The research works discussed by C. Shorten and T. M. Khoshgoftaar in [50] and P. Cao et al., in [51] clarified that employing the augmentation techniques such as geometric transformations, colour space augmentations, kernel filters, mixing images, random erasing, feature space augmentation, adversarial training, neural style transfer, and meta-learning may prove constructive to improve the performance of the DL models. To carry out further research, the authors in [52] conducted the experiments using 124 images downloaded from the Internet. They applied data augmentation techniques such as zoom, rotation, flip, and rescale to increase the size of the dataset to 711 images. In addition, they reported the training accuracy of 95% and the validation accuracy of 89%. The low validation accuracy and impractical implementation using standard memory devices such as mobile phones are the significant limitations of this research. Moreover, they did not consider the parameters such as soil type, temperature, humidity, nutrient requirements, etc. while disease detection. Furthermore, the authors focused only on detecting one disease, ‘downy mildew’ in pearl millet. Therefore, there is considerable scope for improving the performance and working on the most common diseases such as blast and rust.

One more research group in [52] exploited the applications of deep transfer learning. They employed pre-trained VGG-16 [53] to detect downy mildew disease [54] in pearl millet. Based on the experiments, they claimed that deep transfer learning effectively extracts the essential features. The extracted features of the pre-trained network are available for reuse. The pre-trained networks reuse these features and continue learning from the more dataset available for training. This improves the performance of the model. Transfer learning is also important for fine-tuning the model according to the size and type of the dataset. The authors also claimed that transfer learning is helpful to avoid overfitting and to improve the model’s predictive capacity [24].

To work in synergy with the system proposed in [52], the authors in [23,55] integrated IoT and DL techniques for disease detection in crops. However, the system could not prove its practical application due to low accuracy.

The above discussion of the related research works shows that the integration of DL techniques with IoT provides good opportunities for developing the architectures to automate the collection of imagery and parametric data, storage of collected dataset, plant disease detection, generating alerts, and classification of detected diseases. However, these works lack in sensing the parameters that are the root cause for the plant diseases. Moreover, the collection of imagery datasets requires substantial human effort and high cost. Furthermore, the DL models employed for disease detection and classification report low accuracy and take more time to respond. To the best of our knowledge, there is no automatic and intelligent system for identifying and classifying blast and rust diseases in pearl millet. The potential of integration of IoT and deep transfer learning is still underexploited in the field of agriculture.

Therefore, there is a huge scope for improving the performance of the existing systems and providing a new architecture for the automatic collection of the dataset, detection, and classification of diseases in pearl millet.

## 3. Materials and Methods

In this section, the authors present the details of the proposed framework, dataset prepared, training mechanism, and evaluation metrics used to evaluate the performance of the models.

### 3.1. Proposed Framework

**Figure 1:**
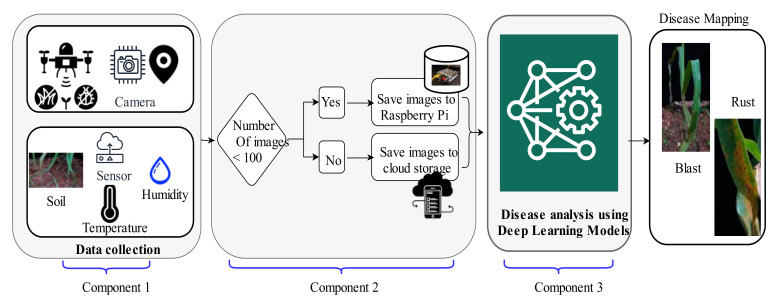
The framework of ‘Automatic and Intelligent Data Collector and Classifier’.

In this manuscript, the authors propose the ‘Automatic and Intelligent Data Collector and Classifier’ (AIDCC) for the collection of data, detection, and classification of rust, and blast diseases in pearl millet. The framework is an integration of three components, as demonstrated in Figure 1.

Component 1: It comprises the digital drone, camera, global positioning system (GPS), and sensors. A digital drone is a crewless aerial vehicle used to monitor the farmlands [56]. Here, the drone is equipped with a Panasonic GH3 camera ‘DJI S1000’ that can focus in the range from 25 to 30 m, offers video resolution of 1920 × 1080, and a CMOS sensor of 16 MP. The camera of the drone clicks the images and transfers them automatically and instantly to the Raspberry Pi and/or cloud storage. It also captures the variation in RGB scaling of plants to spot the major disease areas in the farm. The drone is specified for a flying range of 7 km. The patrolling of the farm using a drone is useful in obtaining the coordinates of the field used by GPS. It saves time and the cost of labor.

In addition to the drone, we used the NIKON D750 digital camera for clicking the pictures. The digital camera is used to capture the desired region such as leaves rather than the complete plant or multiple plants together. Similar to the drone camera, it transfers the captured images directly to the cloud server.

The GPS module embedded with sensors, drones, and Raspberry Pi is helpful to monitor the location of the diseased plants. It is also important to find the region of farmland where fertilizers and/or water are required. The sensors are anchored with the proposed framework to monitor the changes in soil, temperature, and humidity. For example, the variations in temperature and moisture of soil indicate the susceptibility of pearl millet towards the blast and rust diseases [25]. Moreover, the oospores present in the soil are the primary source to infect the underground parts of plants [54]. Therefore, the sensors anchored for continuous monitoring of the soil can detect the presence of oospores in the soil and help in predicting the disease at an early stage. Furthermore, the hyperspectral sensors are fixed with drone cameras for monitoring the environmental and physical conditions.

**Figure 2:**
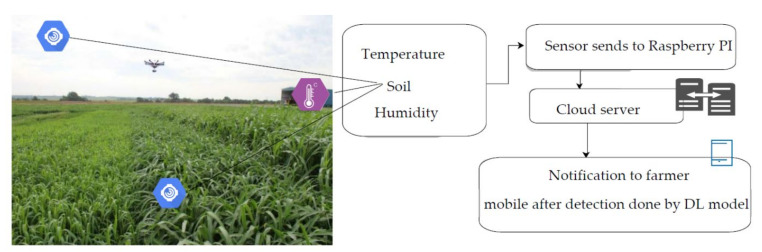
Role of sensors in the pearl millet farmland.

To identify the suitable sensors for the AIDCC, the authors referred to the system proposed in [57]. They used four sensors viz. GY-30, soil sensor, DHT22, and BMP180 sensors to measure the humidity in the soil, temperature, and light intensity. The authors embedded the DS3231 sensor for transferring the information from the mounted sensors to the processor of Raspberry Pi (RPI). Taking clues from the work proposed by N. Materne and M. Inoue in [58] and A. Thorat et al. in [30], we fitted two DHT11 sensors to measure the temperature and humidity in order to spot the rust and blast diseases at an early stage. These sensors are connected to the Raspberry Pi (RPI) for transmitting the captured information to the cloud server. This information is disseminated as an alert or notification to the farmers on their mobile phones. The role of sensors from information gathering to sending notifications to farmers for a pearl millet farmland is demonstrated in Figure 2.

Component 2: This component comprises the Raspberry Pi and cloud storage. It receives the parametric and imagery dataset collected by component 1. Raspberry Pi can store up to 100 images due to its limited storage capacity. Therefore, the photos are sent to the cloud server, if their number exceeds 100, as demonstrated in Figure 1.

Component 3: In this component, the DL based classifier classifies the data stored at the cloud server and Raspberry Pi into the rust and blast classes. Component 3 works synchronously with the Raspberry Pi for facilitating real-time predictions and notifying the farmers about the diseases or other variations observed in the farmland.

### 3.2. Dataset Preparation

For the dataset collection, the hardware components such as drones, digital cameras, and sensors as shown in component 1 of Figure 1, were fixed at the farmland of the Indian Council of Agricultural Research-All India Coordinated Research Project (ICAR (AICRP- Mysore center). The pearl millet plants infected with blast and rust diseases were grown purposefully to monitor the symptoms and impacts of these diseases. The characteristics of diseased leaves of pearl millet are shown in Table 1.

The images of pearl millet plants infected by blast and rust diseases were captured in close observation of the plant pathology expert involved in this research. The authors considered 55- to 60-day-old plants for capturing the images since the blast and rust diseases were easily distinguishable at this age of plants. Moreover, the pathology experts claimed that the degree of severity of rust and blast diseases has reached more than 80% in the plants of this age. In these plants, the pathology experts easily identified rust and blast diseases in pearl millet based on their visible symptoms. For example, the leaves of plants infected with blast turned greyish, and water-soaked lesions appear on the foliage [59]. These lesions vary in size from −2 to 20 mm. The lesions also vary in shape from roundish, elliptical, diamond shaped to elongated. These lesions may enlarge and become necrotic with an increase in the severity of the disease. On the other hand, the leaves of plants infected with rust contain pinhead chlorotic flecks. These flecks turn into reddish-orange as the disease severity increases. Moreover, the round to elliptical pustules appear on both surfaces of leaves [59]. The observable differences in the patterns of both diseases are important for the precise training of the DL model.

**Figure 3:**
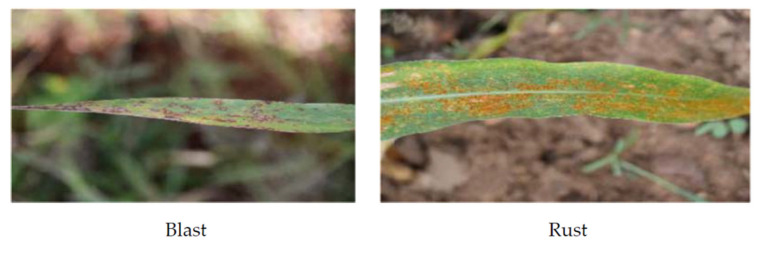
Sample images of pearl millet infected with blast and rust.

The authors captured 1964 images of leaves of pearl millet infected with blast and 1336 images infected with rust. They divided the prepared dataset into training and testing datasets in the ratio of 70% and 30% of the total dataset, respectively. The number of images in these datasets is shown in Table 2, and the sample images of blast and rust diseases are shown in Figure 3.

### 3.3. The Architecture of the ‘Custom-Net’ Model

**Figure 4:**
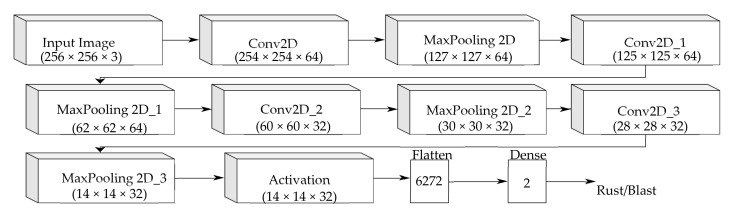
The architecture of the ‘Custom-Net’ model.

The architecture of the ‘Custom-Net’ model designed to predict the samples infected with blast and rust diseases is shown in Figure 4. It comprises four convolution layers, and a max-pooling layer follows each convolution layer. Furthermore, the last max-pooling layer is followed by the activation, flatten, and dense layer.

#### Training of ‘Custom-Net’ and State-of-the-Art Deep Learning Models

Based on the set of experiments conducted and the experimental results reported in the related works [51,60,61], the authors employed the Adam optimizer to deal with the problems of sparse gradients that may be generated on the noisy dataset. This optimizer adopts the best properties of AdaGrad and RMSProp optimization algorithms and favors the better training of the model. Moreover, the authors employed the softmax activation function and categorical cross-entropy loss function for precise training of the proposed network. In addition, they set the learning rate of 0.0001 to optimize the learning of the model and obtain its optimum performance. Furthermore, the authors continuously monitored the model’s performance and observed that it reports the optimum performance for the batch size of 16 samples. 

The authors employed pre-trained and non-pre-trained versions of ‘Custom-Net’ and state-of-the-art models. The model is named as pre-trained if it is trained on the ‘ImageNet dataset’ [62], its weights are saved and it is further trained on the dataset collected in this research. The pre-trained model learns the low-level features such as boundary and edge marking from the ‘ImageNet dataset’. It further learns the high-level features such as pattern differences in leaves infected with blast and rust diseases, from the dataset used in this manuscript. In contrast, the model is named as non-pre-trained if it is initialized with random weights and directly trained with the dataset collected in this research. The non-pre-trained model learns both the high level as well as low-level features from the dataset used in this manuscript.

**Figure 5:**
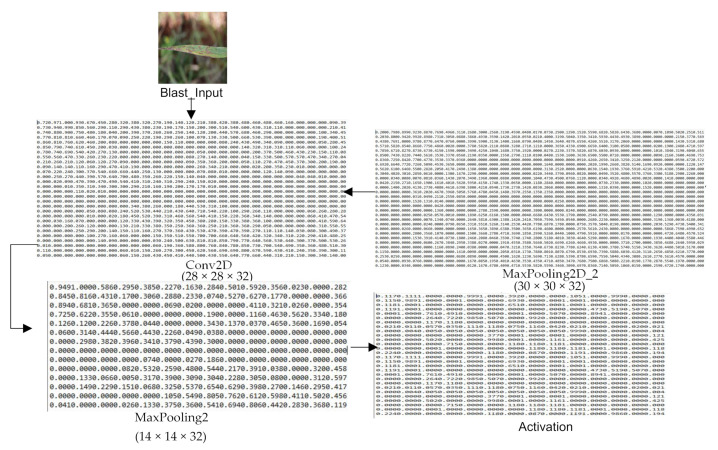
Output matrix of selected layers of ‘Custom-Net’ model.

Now, to showcase the impact of transfer learning on the shallow neural network, the authors pre-trained the ‘Custom-Net’ on the publicly available ‘ImageNet dataset’ comprising more than 14 million images [62] followed by the training on the dataset collected as a part of this research. In addition, they also trained the model only on the collected dataset without using the concept of transfer learning. They compared the results of the pre-trained and non-pre-trained versions of ‘Custom-Net’ to demonstrate the impact of transfer learning on feature extraction and classification. Moreover, they also plotted the output matrix obtained after each layer of the ‘Custom-Net’ as shown in Figure 5. This is important to visualize how the ‘Custom-Net’ extracts the relevant features and ignores the irrelevant features at its different layers. It is evident from Figure 5 that there are no clear boundaries visible at the initial convolution layers. However, the feature map is reduced, and boundaries are more precise at the later convolution layers and their following max-pooling layers. It is apparent from the last matrix shown in Figure 5 that the model learned to identify even the complex patterns hidden in the image.

Moreover, it is clear from the matrices shown in Figure 5 that the model starts learning the pixel-level features at the initial layers. Gradually, it starts discarding the features picked from the background and considers only the relevant features for decision-making once it is trained. They also recorded that each epoch takes 4 s and the model completes its training in 20 epochs. The quick training of the model shows its efficacy in feature extraction.

Now, for comparing the efficacy of the proposed ‘Custom-Net’ model, the authors employed the pre-trained as well as non-pre-trained versions of the state-of-the-art models viz. VGG-16, VGG-19 [53], ResNet-50 [39], Inception-V3 [42], and Inception ResNet-V2 [41] to predict the samples infected with blast and rust diseases.

### 3.4. Evaluation Metrics

To evaluate the performance of the classifiers accompanied by the ‘Automatic and Intelligent Data Collector and Classifier’, the authors used the confusion matrix as presented in [53], average accuracy, precision, recall, and training time. The definitions of these metrics are given below:

Confusion Matrix: This represents the number of correctly and incorrectly classified samples into each labelled class. Here, TB denotes the number of correctly classified samples of blast disease, FB denotes the number of incorrectly classified samples of blast disease, TR is the number of correctly classified samples of rust disease, and FR is the number of incorrectly classified samples of rust disease. The sample confusion matrix is shown in Table 3. Based on the labels presented in the confusion matrix, the authors define the evaluation matrices, namely sensitivity, accuracy, precision, recall, F1 score.

1. Average *accuracy*: It is the measure of the degree of correctness of the classification. It can be calculated using the formula given in Equation (1).
  (1) $$Accuracy=\frac{TB+TR}{TR+FB+FR+TB}$$
2. *Precision*: This is the measure of classifying the samples of the blast correctly to the blast class. The formula to calculate the precision is given in Equation (2).
  (2) $$Precision=\frac{TB}{TB+FB}$$
3. *Recall*: This is the measure of correct identification of samples of the blast class from the total number of samples of that class. The formula to calculate the precision is given in Equation (3).
  (3) $$Recall=\frac{TB\;}{TB+FR}$$

## 4. Results

In this section, the authors present the results obtained by evaluating the performance of the trained ‘Custom-Net’ model on the test dataset comprising 990 images of blast and rust diseases in pearl millet.

### 4.1. Confusion Matrix for Classification

The confusion matrix of the pre-trained ‘Custom-Net’ model on the training and testing datasets are shown in Table 4a,b, respectively. Similarly, the confusion matrix of the non-pre-trained ‘Custom-Net’ model on the training and testing datasets are shown in Table 5a,b, respectively. It is clear from Table 4a and Table 5a that the pre-trained, as well as non-pre-trained models, do not misclassify any sample from the training dataset. Whereas, it is evident from Table 4b that the pre-trained ‘Custom-Net’ model misclassifies 34 samples from the test dataset containing 567 images of plant leaves infected with blast disease. Furthermore, it is clear from Table 4b that the ‘Custom-Net’ model misclassifies 69 images from the test dataset comprising 423 images of plant leaves infected with rust disease. However, at the same time, it can be observed in Table 5b that the non-pre-trained ‘Custom-Net’ model misclassifies only 4 and 8 samples from the testing dataset comprising images of leaves infected with blast and rust diseases, respectively.

**Figure 6:**
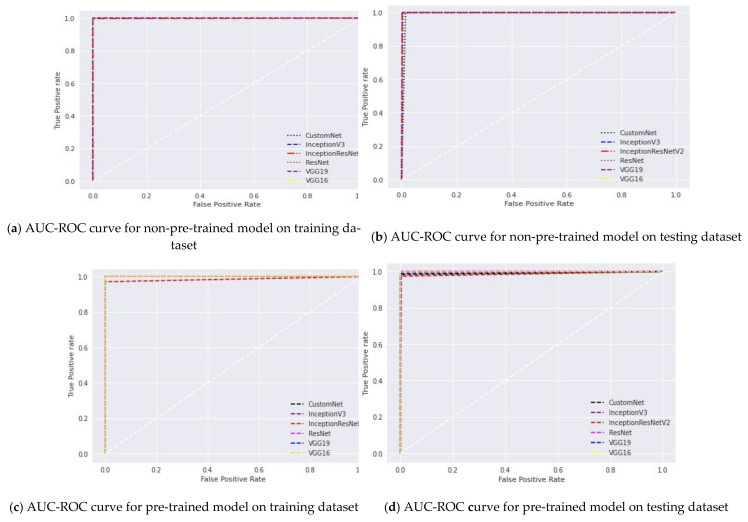
AUC-ROC curves of six classifiers on the training and testing datasets.

Furthermore, it is claimed in [63] that the area under the curve (AUC) and receiver operating characteristic (ROC) (AUC-ROC) curves are the most effective tools for visualizing the classification performance of a model. In this manuscript, these curves are used to check the capability of the model to distinguish the rust and blast disease classes. The AUC-ROC curves for different classifiers on the training and testing datasets are shown in Figure 6.

Furthermore, the authors also present the classification performance of the ‘Custom-Net’ model and state-of-the-art DL models, as shown in Table 6. It is evident from the results shown in Table 6 that except VGG-16 and VGG-19 models, the pre-trained and non-pre-trained versions of all the DL models employed in this manuscript report the equivalent values of accuracy, precision, recall, and F1 score.

### 4.2. Average Accuracy

**Figure 7:**
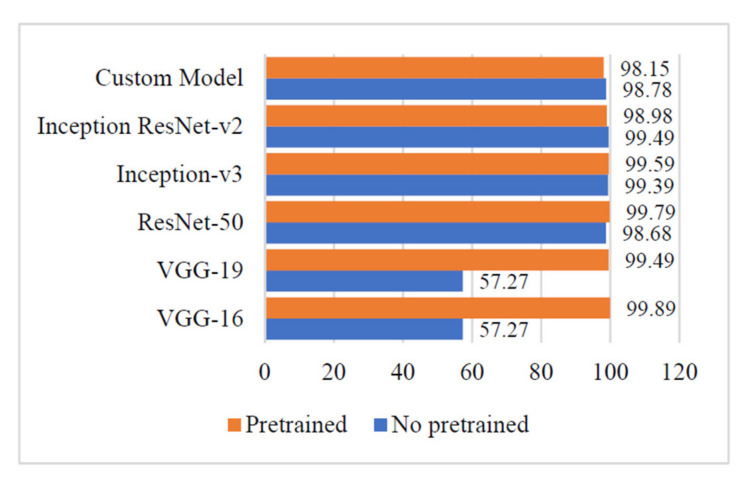
Average accuracy of different deep learning models.

Figure 7 shows that the values of average accuracy, reported by the non-pre-trained and pre-trained versions of the ‘Custom-Net’ and state-of-the-art DL models viz. Inception ResNet-V2, Inception-V3, ResNet-50, VGG-16, and VGG-19, are comparable except for the non-pre-trained versions of VGG-16 and VGG-19.

The Non-pre-trained version of the ‘Custom-Net’ model reported an average accuracy of 98.78%, whereas its pre-trained version reported an average accuracy of 98.15%.

Similarly, the non-pre-trained versions of Inception ResNet-V2, Inception-V3, and ResNet-50 reported the average accuracies of 99.49%, 99.39%, and 98.68%, respectively. It is also apparent from Figure 7 that the pre-trained versions of ResNet-V2, Inception-V3, ResNet-50, VGG-16, and VGG-19 also reported the comparable values of average accuracies of 98.98%, 99.59%, 99.79%, 99.49, and 99.89%, respectively. However, in strong contrast, the non-pre-trained versions VGG-16 and VGG-19 reported a low average accuracy of 57.27%.

### 4.3. Precision

**Figure 8:**
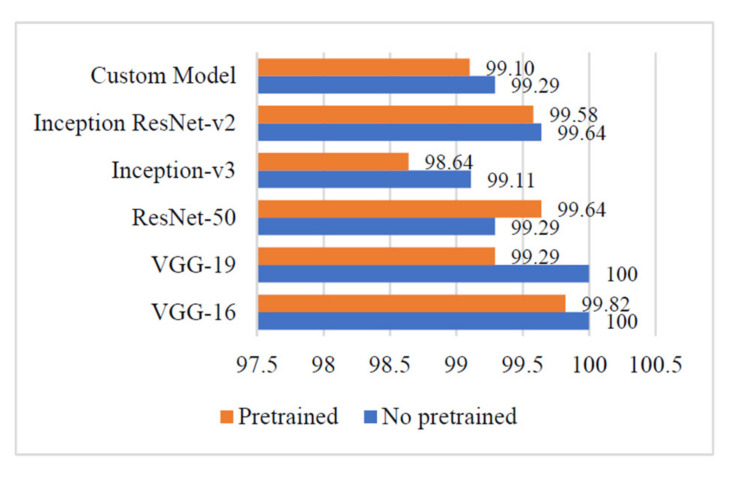
Precision of different deep learning models.

It is evident from Figure 8 that the ‘Custom-Net’ model reported the highest precision of 99.29%. Moreover, there is a slight difference of 0.19% in the precision of its pre-trained and non-pre-trained versions. It is also clear from Figure 8 that the VGG-16 and VGG-19 models reported the highest precision of 100%. There is a minor variation of 0.18% and 0.71% in the precision of the pre-trained and non-pre-trained versions of VGG-16 and VGG-19, respectively. The other deep learning models viz. Inception ResNet-v2, Inception-v3, and ResNet-50 reported the highest precision of 99.64%, 99.11%, and 99.29%, respectively.

### 4.4. Recall

**Figure 9:**
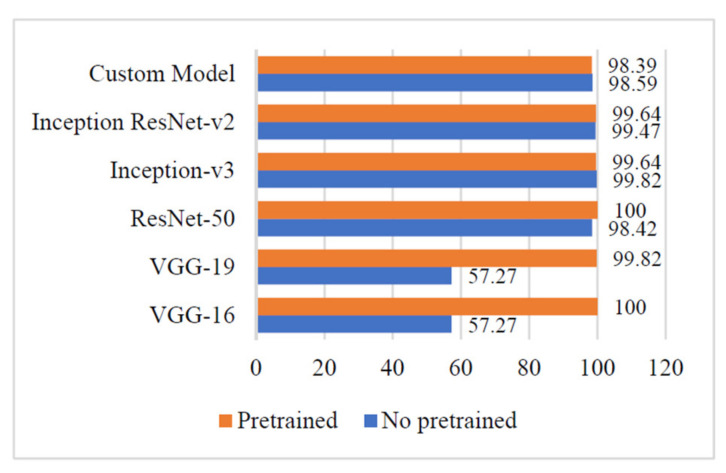
Recall of different deep learning models.

The results shown in Figure 9 indicate that the ‘Custom-Net’ model reported a recall of 98.59%. A minor variation of 0.20% has been observed in the values of recall of its pre-trained and non-pre-trained versions. Additionally, the results also show that VGG-16 and ResNet-50 reported the highest recall of 100%. Moreover, there is a significant variation of 42.73% and 42.55% in the recall of pre-trained and non-pre-trained versions of VGG-16 and VGG-19, respectively. The Inception ResNet-V2, and Inception-V3 reported the highest values of 99.64% and 99.82%, respectively. Furthermore, there is a minor difference of 0.17% and 0.18% in the recall of the pre-trained and non-pre-trained versions of Inception ResNet-V2 and Inception-V3.

### 4.5. F1 Score

**Figure 10:**
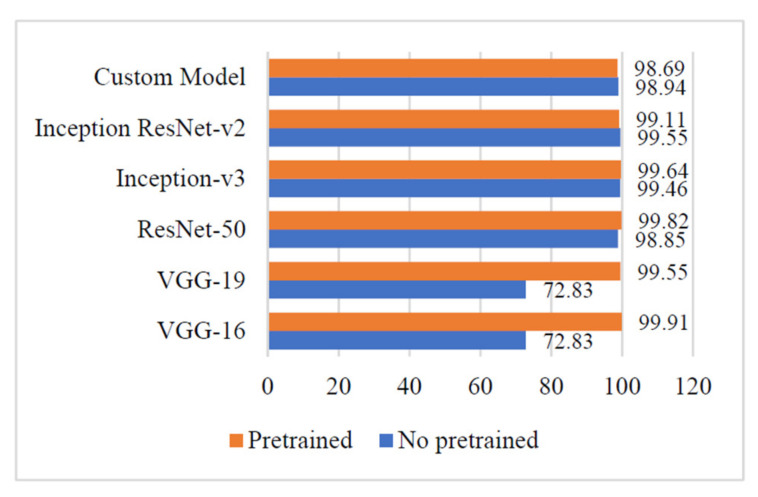
F1 score of different deep learning models.

The experimental results demonstrated in Figure 10 show that the ‘Custom-Net’ model achieved the highest F1 score of 98.94%. It reported a small variation of 0.25% in the F1 score of its pre-trained and non-pre-trained versions. The results shown in Figure 10 also indicate that VGG-16, VGG-19, ResNet-15, Inception-V3, and Inception ResNet-V2, give the highest F1 score values as 99.91%, 99.85%, 99.11%, and 99.64%, respectively. It is also clear from the figure that the VGG-16 and VGG-19 give the highest difference of 27.08% and 26.72%, respectively. There is a minor variation in the F1 score of pre-trained and non-pre-trained versions of ResNet-15, Inception-V3, and Inception ResNet-sV2 models.

### 4.6. Computation Cost

**Figure 11:**
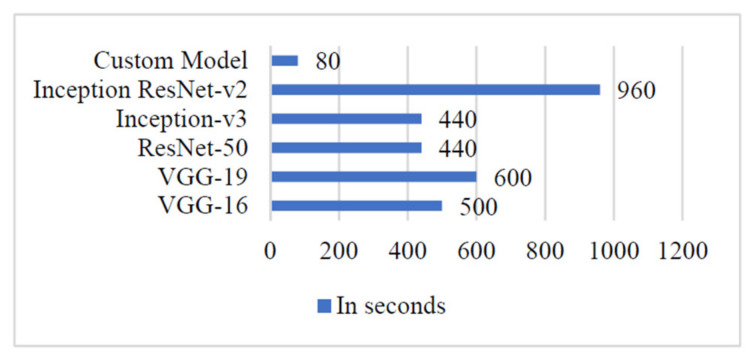
Training time of different deep learning models.

**Figure 12:**
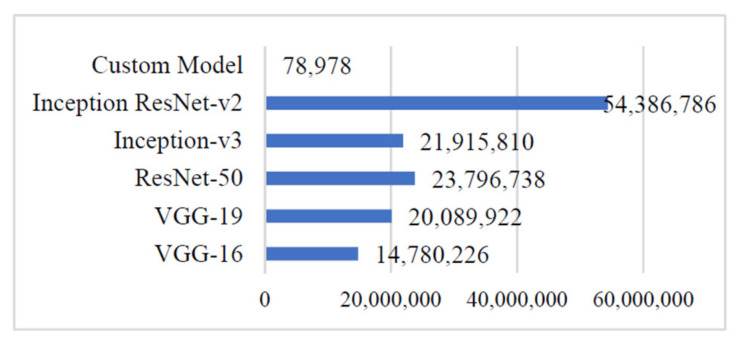
Training parameters of different deep learning models.

To validate the scope of adopting the proposed ‘Custom-Net’ model and state-of-the-art deep learning models viz. Inception ResNet-V2, Inception-V3, ResNet-50, VGG-16, and VGG-19 for classifying diseased leaves, the authors demonstrated the training time and the number of trainable parameters in Figure 11 and Figure 12, respectively. It is noticeable from Figure 12 that the Inception ResNet-V2 model has the maximum number of trainable parameters, whereas the ‘Custom-Net’ model has the minimum number of trainable parameters. Furthermore, it is clear from the training time shown in Figure 11 that the ‘Custom-Net’ model requires a minimum time of only 80 s for training through 20 epochs.

### 4.7. Grad-CAM

**Figure 13:**
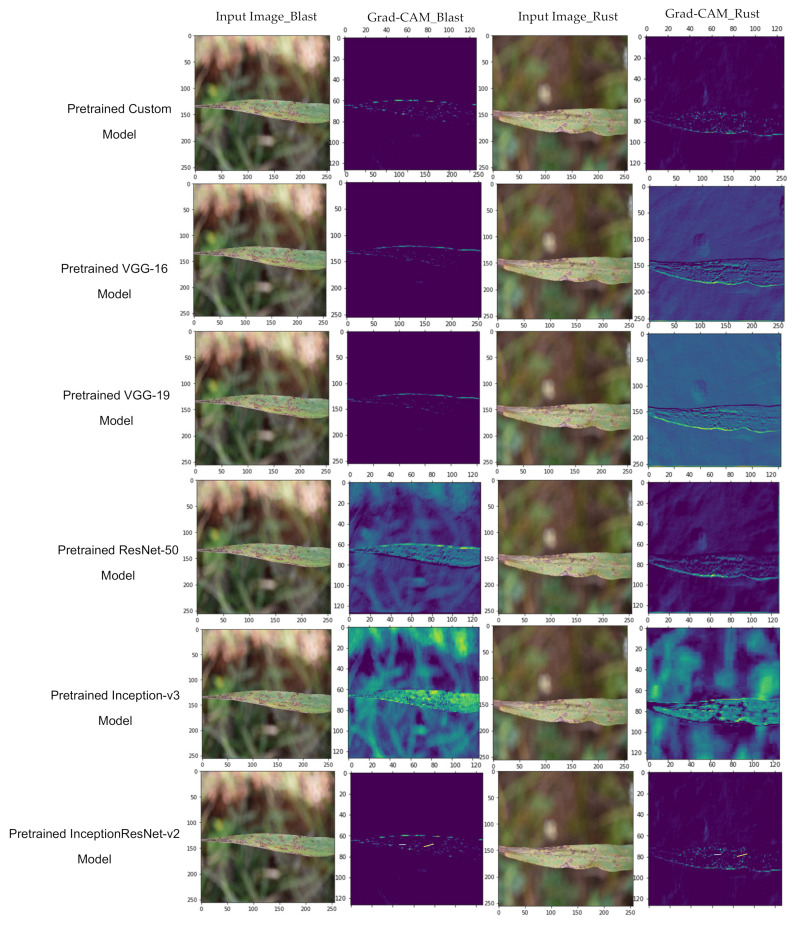
Grad-CAM to visualize the features of pre-trained models.

**Figure 14:**
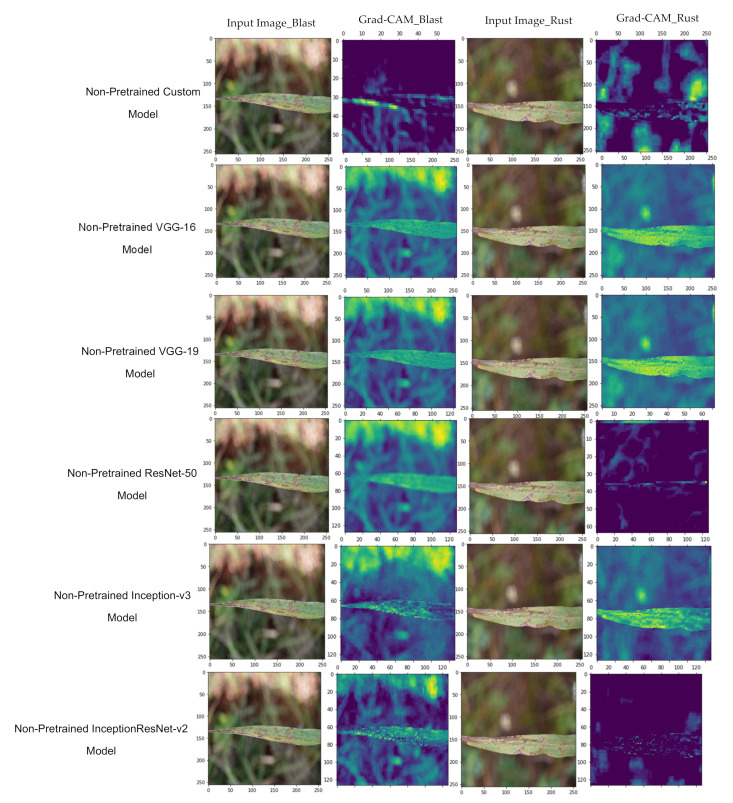
Grad-CAM to visualize the features of non-pre-trained models.

Now, the authors plotted the Grad-CAM to visualize the features involved in the classification. The visualization of features involved in classification for the pre-trained and non-pre-trained versions of Inception ResNet-V2, Inception-V3, ResNet-50, VGG-16, and VGG-19 are shown in Figure 13 and Figure 14, respectively.

## 5. Discussion

In this section, the authors present the inferences deduced from the experimental results obtained by employing the ‘Custom-Net’, Inception ResNet-v2, Inception-v3, ResNet-50, VGG-16, and VGG-19 models.

It is apparent from Figure 6 that the pre-trained version of VGG-16 gives the highest average accuracy. Whereas, the non-pre-trained versions of VGG-16 and VGG-19 reported the minimum value of average accuracy. The pre-training of these models lead to a significant increase of 42.62% in the average accuracy. This proves that these deep networks require a vast dataset for training. Therefore, transfer learning becomes vital for the training of these networks if the dataset size is small. By adopting the advantages of transfer learning, these networks learn the low-level and basic features of the dataset, such as boundary recognition and shape identification. Now, the networks use the weights acquired during pre-training and further learn the recognition of high-level features such as sub-boundaries or details about the image segments.

In contrast, with the models viz. Inception ResNet-v2, Inception-v3s, and ResNet-50, a minor impact of transfer learning was reported on the average accuracy. Similarly, a low impact of 0.63% is observed when the ‘Custom-Net’ model adopted the pre-training and transfer learning.

It is inferred from the above discussion that the shallow neural networks learn the low-level as well high-level features by training on the small dataset size. At the same time, deep networks either require large datasets for training or transfer learning.

Furthermore, the trends of the precision, recall, and F1 measures of the above-stated models are demonstrated in Figure 7, Figure 8 and Figure 9. It is evident from Figure 7 that the non-pre-trained VGG-16 and VGG-19 models reported the highest precision of 100%. In contrast, the non-pre-trained Inception-V3 model gave the lowest precision of 99.11%. The other non-pre-trained models viz. Inception ResNet-v2, ResNet-50, and ‘Custom-Net’ reported 0.36%, 0.71, and 0.71% lower precision than the VGG-16 and VGG-19 models. The small variation in the precision of all the non-pre-trained versions of the above-stated models implies that these models are efficient in recognizing the relevant instances of each class from the input test dataset.

The discussion proves that both the pre-trained and non-pre-trained models are efficient in recognizing the relevant instances of each class from the input test dataset. Moreover, the pre-training helps discriminate the relevant and irrelevant features.

A further analysis of the results shown in Figure 8 reveals that the pre-trained VGG-16 and ResNet-50 models reported a 100% recall. The other pre-trained models Inception ResNet-v2, Inception-v3, VGG-19, and ‘Custom-Net’ reported the 0.61%, 0.36%, 0.18, and 1.61% lower values of recall, respectively.

It is evident from the comparison of non-pre-trained models that the Inception-v3 reported the highest value of 99.82% recall. The other models viz. Inception ResNet-v2, ResNet-50, and ‘Custom-Net’ also reported the equivalent values of recall, as shown in Figure 8. Moreover, there is a minor difference of 0.17% and 0.18% in the recall of the pre-trained and non-pre-trained versions of Inception ResNet-V2 and Inception-V3. Therefore, the comparable values of recall for all the above-stated models indicate that all the models are efficient in correctly identifying the blast disease from the leaves of pearl millet.

However, the VGG-16 and VGG-19 models gave the lowest values of 57.27% recall. Moreover, there is a significant variation of 42.73% and 42.55% in the recall of pre-trained and non-pre-trained versions of VGG-16 and VGG-19, respectively.

This proves that transfer learning is important for the deeply layered models such as VGG-16 and VGG-19, in order to minimize the number of misclassification of leaves infected by blast disease to the rust class.

Moreover, it is evident from the F1 score shown in Figure 9 that the pre-trained VGG-16 model reported the highest F1 score of 99.91%. Furthermore, the other models viz. Inception ResNet-v2, Inception-v3, VGG-19, and ‘Custom-Net’ reported the equivalent values of the F1 score.

Simultaneously, it is also observed that the models, viz. ‘Custom-Net’, ResNet-50, Inception-V3, and Inception ResNet-V2 reported the slight variations of 0.25%, 0.44, 0.18%, and 1.03%, respectively in the F1 score of their pre-trained and non-pre-trained versions. However, the VGG-16 and VGG-19 gave the highest difference of 27.08% and 26.72%, respectively. This proves that transfer learning is important for relevant feature extraction and minimizing the number of misclassifications in VGG-16 and VGG-19 models. However, there is an insignificant impact on the performance of the other above-stated state-of-the-art models. Moreover, the comparable values of the F1 score of all the models reflect that these models are efficient in correctly identifying the samples of the blast as well as rust diseases from the test dataset.

Furthermore, it is coherent from the Grad-CAM plotted in Figure 10 and Figure 11 that the pre-trained ‘Custom-Net’ model is effective in recognizing the acceptable boundaries from the leaves infected with blast and rust. Therefore, it makes the classification based on the relevant features rather than noise. In contrast, its non-pre-trained version is efficient in identifying all the relevant features. Still, it also picks some features from the background that may increase the number of misclassifications.

Similarly, the pre-trained versions of the above-stated state-of-the-art models also perform as a better feature extraction than their non-pre-trained versions. This proves that transfer learning helps the model in the extraction of more relevant features, recognizing acceptable boundaries, and preventing the involvement of noise in decision-making.

However, the ‘Custom-Net’ model shows comparable values of average accuracy, precision, recall, and F1 score with the state-of-the-art models, but there is a significant decrease in the number of trainable parameters and training time. It is noticeable from Figure 11 that the ‘Custom-Net’ model has the minimum number of trainable parameters. Moreover, it is evident from the training time presented in Figure 12 that the ‘Custom-Net’ model requires a minimum time of 4 s per epoch. It completes its training in merely 80 s through 20 epochs. The analysis of training time shows that it requires 84%, 86.6%, 81.81%, 81.81%, and 91.67% lower training time than VGG-16, VGG-19, ResNet-15, Inception-V3, and Inception ResNet-V2 models, respectively. Its efficacy in achieving the classification accuracy comparable to the state-of-the-art models and low training time proves its usability for real-life systems. Furthermore, it is effective in quick decision-making to classify the blast and rust diseased samples in real-time. Table 7 presents the comparative analysis of the approaches available in literature and the approach proposed in this manuscript. 

Moreover, the technique has a biological significance too. The quick and automatic detection of plants infected with rust and blast helps the farmers apply disease control measures, thus preventing the further spread of diseases to the whole farmland. 

To further validate the efficacy of the proposed model ‘Custom-Net’, the authors compared its performance with the state-of-the-art models viz. VGG-16, VGG-19, ResNet-15, Inception-V3, and Inception ResNet-V2. The comparison shows that the ‘Custom-Net’ model efficiently extracts relevant features and involves the relevant features in the decision-making. It achieved the classification performance equivalent to the InceptionResNet-V2. Moreover, it requires a minimum time for training. Therefore, the authors integrated the ‘Custom-Net’ model in the ‘Automatic and Intelligent Data Collector and Classifier’.

In the future, there is a scope of making the predictions based on the parametric dataset collected by the data collector part of the proposed framework. Moreover, there is a need to develop a multi-class classifier to classify the healthy plants infected with Downey mildew, blast, smut, ergot, and rust.

## 6. Conclusions

The framework ‘Automatic and Intelligent Data Collector and Classifier’ (AIDCC) is proposed in this manuscript for automating the collection of imagery and parametric datasets from the pearl millet farmland, feature visualization, and prediction of blast and rust diseases. The framework is an appropriate integration of IoT and deep learning to analyze imagery and numeric data. The hardware components, such as drone cameras, digital cameras, sensors, etc. are anchored in the pearl millet farmland at ICAR, Mysore, India, to collect data automatically. The ‘Custom-Net’ model is designed as a part of this research and deployed on the cloud server. This DL model processes the data collected by the data collector and provides real-time prediction for the blast and rust diseases in pearl millet. Moreover, to showcase the impact of transfer learning, the authors pre-trained the proposed model on the online available ImageNet dataset. The pre-trained model is further trained on the dataset of 2310 images of leaves of pearl millet infected with blast and rust. The performance of the pre-trained and non-pre-trained ‘Custom-Net’ models is evaluated. Based on the visualization of features through Grad-CAM, it is concluded that transfer learning improves the extraction of relevant features and helps the model discard the features picked from the background. At the same time, the slight difference of 0.25% in the F1 score of pre-trained and non-pre-trained ‘Custom-Net’ models prove that being a shallow network, it is equally efficient in making correct classifications even though the training dataset is small.

Moreover, the authors compared the performance of the pre-trained and non-pre-trained state-of-the-art DL models viz. VGG-19, VGG-16, Inception ResNetV2, Inception V3, and ResNet-50 architectures. Furthermore, the authors implemented these models using transfer learning. They employed the pre-trained models on the ImageNet dataset and further trained them on the dataset collected by the data collector of the framework proposed in this research. However, the pre-trained and fine-tuned VGG-19 model outperformed all the models. It achieved the highest values of 99.39%, 99.82%, 99.11%, and 99.46% for the average accuracy, precision, recall, and F1 score, respectively, on the test dataset comprising 990 images of leaves infected with blast and rust. However, this model requires a training time of 600 s that is 86.67% higher than the ‘Custom-Net’ model. Moreover, the high number of training parameters of 20,089,922 increases its computation cost. Therefore, the authors deployed the pre-trained and fine-tuned ‘Custom-Net’ model as a classifier in the framework ‘AIDCC’. Therefore, this research provides a low-cost and user-friendly framework for automating the data collection, feature visualization, disease detection, and prediction of blast and rust diseases in pearl millet. As a result, it may prove a significant contribution to the food industry and farmers in order to increase the yield and quality of crop products.

## Figures and Tables

**Table 1:** Characteristics and symptoms of diseased leaves of pearl millet.

Name of Disease	Causing Agent	Stage of Infection	Shape of Infected Region	Colour of Infected Region
Downy mildew	*Sclerospora graminicola*	Seedling.	Foliar and green ear	Green and whitish
Blast	*Magnaporthe grisea*	Seedling and tillering stage	Elliptical or diamond-shaped	Pale green to greyish green, later turning yellow to grey with age
Rust	*Puccinia substriata var. indica.*	Before flowering	Pistules type small spots	Reddish-orange

**Table 2:** Number of images in training and testing datasets of blast and rust.

Name of Disease	Total Number of Images	Number of Images in the Training Dataset	Number of Images in Testing Dataset
Blast	1964	1375	567
Rust	1336	935	423
Total	3300	2310	990

**Table 3:** Sample Confusion matrix.

Actual Label			
Predicted Label		Blast	Rust
	Blast	TB	FB
	Rust	FR	TR

**Table 4:** Confusion matrix of pre-trained ‘Custom-Net’ model.

(**a**) Training dataset			
	Actual Label		
Predicted Label		Blast	Rust
	Blast	1375 (TB)	0(FB)
	Rust	0(FR)	935 (TR)
(**b**) Testing dataset			
	Actual Label		
Predicted Label		Blast	Rust
	Blast	533 (TB)	34(FB)
	Rust	69(FR)	354(TR)

**Table 5:** Confusion matrix of non-pre-trained ‘Custom-Net’ model.

(**a**) Training dataset			
	Actual Label		
Predicted Label		Blast	Rust
	Blast	1375	0
	Rust	0	935
(**b**) Testing dataset			
	Actual Label		
Predicted Label		Blast	Rust
	Blast	563 (TB)	4(FB)
	Rust	8(FR)	415(TR)

**Table 6:** Classification performance of different deep learning models.

Metrics	Non-Pre-Trained Models						Pre-Trained Models					
	VGG-16	VGG-19	ResNet-50	Inception-V3	Inception ResNetV2	‘Custom-Net’	VGG-16	VGG-19	ResNet-50	Inception-V3	Inception ResNetV2	‘Custom-Net’ Model
Accuracy (%)	57.27	57.27	98.68	99.39	99.49	99.78	99.89	99.49	99.79	99.59	98.98	98.15
Precision (%)	100	100	99.29	99.11	99.64	99.29	99.82	99.29	99.64	98.64	99.58	99.10
Recall (%)	57.27	57.27	98.42	99.82	99.47	98.59	100	99.82	100	99.64	99.64	98.39
F1 score (%)	72.83	72.83	98.85	99.46	99.55	98.94	99.91	99.55	99.82	99.64	99.11	98.69

**Table 7:** Comparison of the proposed approach and the approaches available in literature.

Reference	Year	Crop	Diseases	Number of Images, Source	Tools Used for Dataset Collection	Model(s) Applied	Evaluation Metrics
Our work	2021	Pearl millet	Rust, blast	3300, ICAR Mysore	X8-RC Drone camera NIKON D750 Digital camera DHT11 sensor Raspberry Pi	‘Custom-Net’ VGG-16 VGG-19 ResNet-50 Inception-v3 Inception ResNet-v2	Accuracy = 98.78% Precision = 99.29% Recall = 98.59% F1 score = 98.64% Training time = 80 s Number of training parameters = 78,978
[28]	2020	Tomato	Early blight Late blight Healthy	5923 Plant Village Dataset, Internet images, and leaf images captured from Tansa Farm, Bhiwandi	Sensor	Support vector machines Random Forest (RF) K-means VGG-16 VGG-19	Clustering accuracy using RF = 99.56% Classification accuracy using VGG-16 = 92.08%
[23]	2020	59 categories	49 disease categories, 10 healthy	36,252, AI-challenger	Video cameras Smartphone	MDFC-ResNet VGG-19 AlexNet ResNet = 50	Accuracy = 93.96% Precision = 98.22% Recall = 95.40% F1 score = 96.79%
[52]	2019	Pearl millet	Downy mildew	711 Images from the Internet	No camera No IoT	VGG-16 Transfer learning	Accuracy = 95% Precision = 94.50% Recall = 90.50% F1 score = 91.75%
[29]	2018	Rice	Bacterial Blight Sheath Blight Brown Spot Leaf Blast	International Rice Research Institute (IRRI) database	Drone Camera GPS sensor	Support vector machine (SVM)	Only disease boundary detected

## Data Availability

Data prepared as a part of this research is available at https://www.kaggle.com/kalpitgupta/blast-and-rust-compressed. The researchers who wish to use the dataset available at the above link must cite this article.
